# Viologen-Directed Silver-Thiocyanate-Based Photocatalyst for Rhodamine B Degradation in Artificial Seawater

**DOI:** 10.3390/ma17215289

**Published:** 2024-10-30

**Authors:** Xueqiang Zhuang, Xihe Huang, Haohong Li, Tianjin Lin, Yali Gao

**Affiliations:** 1 School of Marine Engineering, Jimei University, Xiamen 361021, China; xqz@jmu.edu.cn (X.Z.); 201861000071@jmu.edu.cn (T.L.); 2 Fujian Province Key Laboratory of Ship and Ocean Engineering, Xiamen 361021, China; 3 Fujian Institute of Innovation for Marine Equipment Detection and Remanufacturing Industrial Technology, Xiamen 361021, China; 4 College of Chemistry, Fuzhou University, Fuzhou 350116, China; xhhuang@fzu.edu.cn (X.H.); lihh@fzu.edu.cn (H.L.)

**Keywords:** pollutant treatment in sea, viologen/silver-thiocyanate hybrid, crystal structure, photocatalytic degradation

## Abstract

Photocatalytic degradation is a leading technology for complete mineralization of organic dyes in the ocean. In this work, a new viologen-bearing silver-thiocyanate-based photocatalyst, i.e., {(*i*-PrV)[Ag_2_(SCN)_4_]}*_n_* (*i*-PrV^2+^ = isopropyl viologen) has been synthesized and structurally determined, with results showing that it can exhibit excellent degradation performance on rhodamine B (RhB) in artificial seawater. The planar *i*-PrV^2+^ dications are confined in the free voids of the [Ag_2_(SCN)_4_]*_n_*^2*n*−^ layer with a two-dimensional (6,3) mesh, and strong C-H···S hydrogen bonds contribute to its structural stabilization. This photocatalyst was further characterized by powder X-ray diffraction (PXRD), UV-Vis, fluorescence, and photo/electrical responsive measurements, pointing to its application in visible-light-driven catalysis. Interestingly, using this photocatalyst, good photocatalytic degradation performance on rhodamine B in artificial seawater could be observed. The dye pollutant could be degraded with a high degradation ratio of 87.82% in 220 min. This work provides a promising catalyst for organic dye-type ocean pollutant treatments.

## 1. Introduction

Over the past two decades, artificial compound-type hazardous substances have greatly threatened the seawater ecosystem [1]. Organic pollutants, including synthetic dyes, are classified as the most dangerous because they are persistent in the environment [2], and are divided into azo, acid, basic, and other reactive dyes [2,3]. Among these, rhodamine B (RhB) is one of the oldest dyestuffs commonly used in the food and fabric industries, and is difficult to biochemically degrade and results in serious environment problems with biological toxicity [4,5]. This pollutant can flow into rivers and accumulate in the sea; for example, a recent study reported that dye pollutants can be found in the muscle tissue of European eel [6]. Thus, the degradation of RhB residues in seawater seems significant. Photocatalytic degradation is a leading technology for complete mineralization of organic dyes. TiO_2_-based semiconductors were first adopted in photocatalytic treatment [7,8]. In the last two decades, versatile photocatalysts have emerged, including nano zinc oxide (ZnO) [9,10], CdS [11], 2D PbMoO_4_ [12,13], g-C_3_N_4_ [14,15], and crystalline inorganic/organic hybrids [16,17,18,19,20]. However, until now, most of the work on dye degradation has been executed in freshwater, and research on degradation of dye in saline solutions is still very rare [21,22,23,24,25,26,27]. Chemical dye is a bulk commodity transported by marine transportation, and the risk of an accident at sea is high. Therefore, research about dye degradation in seawater is urgently needed. Here, salinity, including Na^+^, Mg^2+^, and Ca^2+^ cations in seawater, affects the degradation efficiency [28]. Crystalline inorganic/organic hybrids are special due to their tunable pore sizes and electronic characters with distinct structures [16]. For example, lead iodide/viologen hybrids, i.e., (PBPY-H_2_)_2_[PbI_4_(I_3_)_2_]), can degrade RhB driven by visible light in water [18,19]. They have demonstrated stable and high photocatalytic efficiency for RhB degradation in seawater [24]. Their distinct structures are beneficial for the discovery of the mechanism. Recently, viologens (*N*,*N′*-disubstituted 4,4’-bipyridinium) have attracted great interest due to their excellent electron-accepting ability [29,30], and have exhibited potential applications in dye degradation [18,24]. In this work, viologen and silver-thiocyanate were incorporated to generate a stable inorganic/organic hybrid (i.e., {(*i*-PrV)[Ag_2_(SCN)_4_]}*_n_*, *i*-PrV^2+^ = isopropyl viologen), which served as a highly efficient photocatalyst in RhB degradation in seawater.

## 2. Experimental

### 2.1. Materials and Methods

*i*-PrV·(NO_3_)_2_ (*i*-propyl viologen nitrate) was self-prepared. Other reagents with AR quality were obtained from commercial sources and used without further purifications. A Nicolet Co. (Nicolet Co., Madison, WI, USA) Magna-IR 750 spectrometer was adopted for IR measurement with KBr pellets in the scanning range of 4000–400 cm^−1^. A Vario MICRO elemental analyzer was utilized to conduct elemental analyses. Upon Cu Kα radiation with a wavelength of 1.54056 Å, powder X-ray diffraction (PXRD) patterns were captured on a Philips X’Pert-MPD diffractometer (Panalytical Co., Amsterdam, The Netherlands). Adsorption performance characterized by UV-vis spectra (200–800 nm) was recorded on a PerkinElmer (Waltham, MA, USA) lambda 900 UV/vis spectrophotometer. The K–M equation, i.e., α/S = (1 − R)^2^/(2R) was adopted to calculate the optical edges [31]. The N_2_ adsorption–desorption isotherm was measured on a BET ASAP 2020 PLUS analyzer, based on which the surface area was calculated by the BET algorithm. The photocurrent (I-t curve) was collected on an electrochemistry workstation (CHI650). Contact angle (CA) was measured on a JC2000C machine using 4 μL water (Powereach, Shanghai, China).

### 2.2. Synthesis

**Scheme 1:**
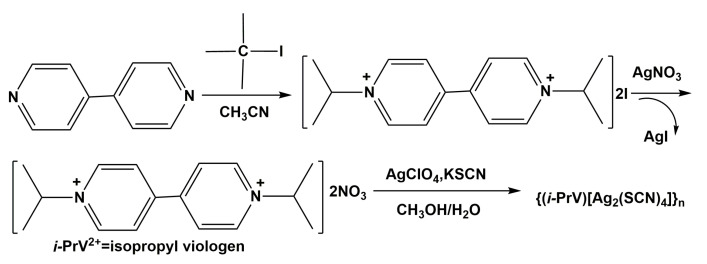
Synthesis of *i*-PrV·(NO_3_)_2_ and photocatalyst.

**Synthesis of *i*-PrV·(NO_3_)_2_:** *i*-PrV·(NO_3_)_2_ was prepared according to the literature method (Scheme 1) [32]. 4,4′-bipyridine (3.12 g, 0.02 mol) was dissolved in 50 mL acetonitrile, and 2-iodopropane (10.20 g, 0.06 mol) was added slowly dropwise and refluxed at 70 °C for 12 h. A large amount of yellow powder was precipitated in a round bottom flask. The resultant mixture was cooled to room temperature and filtered. *i*-PrV·I_2_ (4.66 g, yield: 47%) was obtained, which was dried under vacuum. Afterwards, *i*-PrV·I_2_ (4.96 g, 0.01 mol) was completely dissolved in 50 mL distilled water to obtain a yellow solution, and then AgNO_3_ (3.40 g, 0.02 mol) was dissolved in 20 mL of distilled water in another beaker. The above solutions were mixed and stirred to produce a large amount of yellow AgI precipitates, which were filtered to obtain a colorless and transparent liquid. The filtrate was evaporated to obtain a pale product of *i*-PrV·(NO_3_)_2_ (2.00 g, yield: 54%).

**Synthesis of {(*i*-PrV)[Ag_2_(SCN)_4_]}*_n_*:** *i*-PrV·(NO_3_)_2_ (0.0366 g, 0.1 mmol) was dissolved in 5.5 mL CH_3_OH/H_2_O mixed solvent (volume ratio: 4.5:1), which was stirred for 15 min. In another beaker, AgClO_4_ (0.0207 g, 0.1 mmol) and KSCN (0.038 g, 0.4 mmol) were dissolved in 4.5 mL CH_3_CN, which was stirred at room temperature for 30 min. After mixing the above two solutions for 30 min, its pH value was set at 5–6 with 70% HClO_4_. The mixture was stirred for an additional 2 h; afterwards, a yellow transparent solution was obtained by filtering. After 10 days, yellow block crystals were precipitated in the beaker. About 0.0475 g of crystals were collected by filtration, and the yield was 68.8% (based on Ag). Anal. Calc. For {(*i*-PrV)[Ag_2_(SCN)_4_]}*_n_* (345.21): calcd: C 34.79, H 3.21, N 12.17; found: C 34.76, H 3.19, N 12.18. IR (KBr, cm^−1^): 3127(w), 3058(w), 2989(w), 2143(m), 2083(s), 1632(m), 1559(m), 1501(m), 1447(m), 1375(m), 1318(w), 1255(m), 1166(m), 1139(m), 1080(m), 1046(w), 900(w), 818(s), 742(m), 713(m), 529(m).

### 2.3. X-Ray Crystallography

During the single crystal X-ray diffraction measurement on a Bruker (Bruker Co., Karlsruhe, Germany) APEX II diffractometer, the wavelength of 0.71073 Å was obtained from a graphite-monochromated MoKα radiation. The crystal structure was solved by direct methods, during which the full-matrix least-squares technique on *F*^2^ was adopted with the SHELXL-2018/3 program in OLEX-2 [33,34]. Anisotropic refinements were applied on all non-hydrogen atoms, and hydrogen atoms were geometrically placed. The crystallographic parameters and refinement details are given in Table 1, selected bond lengths/angles are shown in Table 2, and hydrogen bonds can be found in Table 3. CCDC 2289246 contains the supplementary crystallographic data for this paper, which can be obtained free of charge via www.ccdc.cam.ac.uk/data_request/cif, or by emailing data_request@ccdc.cam.ac.uk.

### 2.4. Photocatalytic Testing

Artificial seawater was self-prepared according to the literature. In detail, the components were as follows: NaCl (77.9%), MgCl_2_ (9.6%), MgSO_4_ (6.1%), CaSO_4_ (4.0%), and KCl (0.07%) [35]. Photocatalytic degeneration on RhB in seawater was conducted at an ambient atmosphere. In 80 mL seawater with a RhB concentration of 10 ppm, 50 mg catalyst powder was dispersed. Adsorption/desorption balance must be first achieved, for which the suspension was stirred in the darkness for 2 h. Then, a 300-W Xe arc lamp was opened and maintained irradiation for 220 min, which served as a light source. A quantity of 3 mL of the liquid sample was taken from the irradiated solution at intervals of fixed time, and was treated by centrifugal separation at once. In the meantime, UV−Vis measurements were executed on these degenerated samples, and consequently, their residual concentrations in solution were determined. Herein, the C/C_0_ was calculated as the degradation percentages, wherein C was the absorption intensity at 553 nm of RhB of each sample, and C_0_ was the starting intensity using the first sample.

## 3. Results

### 3.1. Crystal Structure of Catalyst

**Figure 1:**
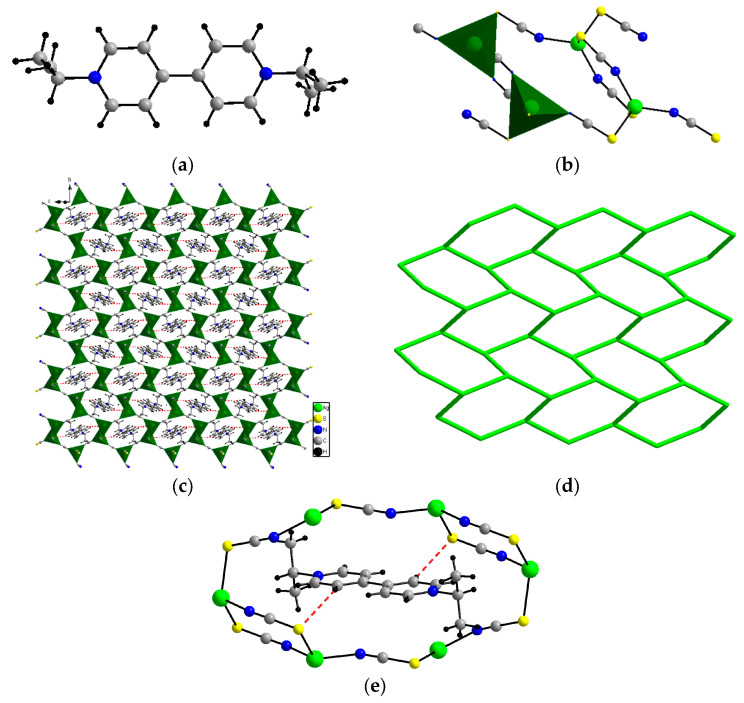
(**a**) Structure of *i*-PrV^2+^ cation; (**b**) structure of [Ag_4_(SCN)_10_] unit; (**c**) 2-D image of {(*i*-PrV)[Ag_2_(SCN)_4_]}*_n_*; (**d**) the (6,3) net topology of [Ag_2_(SCN)_4_]*_n_*^2*n*−^ layer; (**e**) C-H···S hydrogen bonds between [Ag_2_(SCN)_4_]*_n_*^2*n*−^ layer and *i*-PrV^2+^ cation.

{(*i*-PrV)[Ag_2_(SCN)_4_]}*_n_* belongs to a monoclinic system in *P*2_1_/*c* space group. In *i*- PrV^2+^ dications, the dihedral angle defined by two pyridine is close to zero, indicating its good planarity (Figure 1a). The inorganic [Ag_2_(SCN)_4_]*_n_*^2*n*−^ layer is similar to that of {(EV)[Ag_2_(SCN)_4_]}*_n_* [20]. The Ag center adopts the distorted tetrahedral geometry (Ag(1)N_2_S_2_ pattern) by two N and S atoms (Figure 1b, Table 2). Two Ag(1)N_2_S_2_ units are combined into a Ag_2_SCN_6_ dimer through two bridging SCN^−^ ions (Figure 1b). Through four bridging SCN^−^ ions, these dimers are linked into a 2D layer (Figure 1c). The [Ag_2_(SCN)_4_]*_n_*^2*n*−^ layer can also be described as hexagonal, consisting of two Ag_2_(SCN)_2_ rectangular motifs and four *μ*_2_-SCN^−^ anions, whose topology could be depicted as a 2D (6,3) mesh (Figure 1d). Among these hexagonal rings, the size of the elliptical endopore-like pore is 1.538 × 0.948 nm^2^. The Ag···Ag distance separated by the *μ*_2_-NCS bridge is 6.340(2) Å, while that in the Ag_2_(SCN)_2_ cell is 5.584(4) Å. These distances rule out the presence of argentophilic interactions in this hybrid. The S···S distance in the neighboring layers is enough so that there is no S···S interaction. As shown in Figure 1e, *i*-PrV^2+^ is confined in the cavity by C-H···S hydrogen bonding, and layer accumulation is further enhanced by the hydrogen bonding through *μ*_2_-NCS (Table 3).

### 3.2. Characterizations and Photocurrent Response Behavior of Catalyst

**Figure 2:**
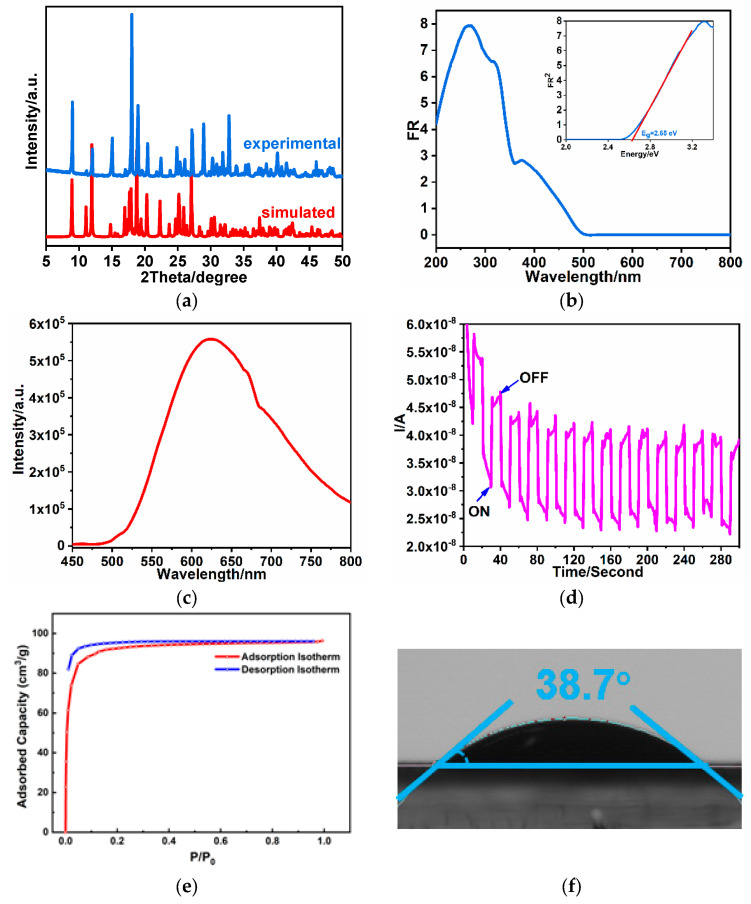
Characterizations of {(*i*-PrV)[Ag_2_(SCN)_4_]}*_n_*: (**a**) simulated and experimental PXRD patterns; (**b**) solid state adsorption spectrum (inserted: optical gap); (**c**) solid state fluorescence spectrum (λ_ex_ = 430 nm); (**d**) photocurrent response behavior; (**e**) N_2_ adsorption–desorption isothermal curve; (**f**) water contact angle.

The photocatalyst {(*i*-PrV)[Ag_2_(SCN)_4_]}*_n_* was characterized by PXRD and UV-Vis spectra. As suggested by PXRD, the experimental diffraction patterns were consistent with the simulated ones in X-ray single crystal structure determination, verifying their good purity (Figure 2a). The photo-response region was determined by the solid state diffuse-reflectance UV-Vis spectrum of the as-synthesized sample (Figure 2b). The peaks at 267 nm stem from the π-π* transitions of the pyridine ring in the viologen [36]. The broad adsorption bands in the range of 300~500 nm originate from the charge transfer from electron-rich silver-thiocyanate (as donors) to viologen (acceptors) [37]. Based on the K-M equation, its optical gap is estimated as 2.65 eV, rendering its semiconductor nature. The adsorption in the visible zone and appropriate optical gap hint at its application as a visible-light-driven catalyst in dye degeneration. The solid photoluminescence spectrum suggests its strong red emission at 624 nm upon exciting at 430 nm, which can be assigned to the metal-to-ligand charge transfer (MLCT, i.e., the electron transfer from the Ag center to π anti-bonding orbitals of SCN^−^) (Figure 2c) [20]. The charge separation efficiency of this photocatalyst was estimated by photoelectrical responsive measurement. The photocatalyst-coated ITO electrode was prepared by the solution method [29]. In the photocurrent measurement, a 150 W xenon lamp with an ON–OFF interval of 20s was utilized. Upon illuminating the electrode, repeatable photocurrents could be found (photocurrent density: 4.0 × 10^−8^ A), which implies the good charge-separation efficiency of this photocatalyst (Figure 2d). This photocatalyst is hydrophilic, which is validated by its water contact angle of 41.8° (Figure 2f).

### 3.3. Photocatalytic Degradation of RhB in Seawater

**Figure 3:**
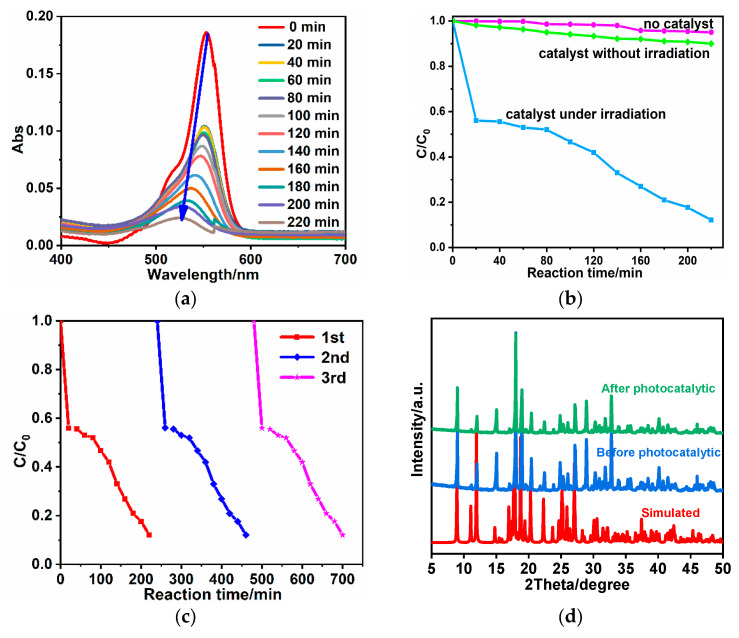
(**a**) UV-Vis spectra of seawater containing RhB and catalyst at different irradiating times; (**b**) degradation ratio of RhB and reference experiments; (**c**) recycling performance of photocatalyst for RhB photodegradation; (**d**) PXRD patterns of photocatalyst before and after photocatalytic reaction.

Rhodamine B is the most frequently used dye in fiber or paint fields, but it is also toxic to living organisms and the environment. The near-visible light adsorption, low optical gap, and good charge separation efficiency imply its potential photocatalytic activity. During the photocatalytic measurement, 3 mL aliquots of reaction system were extracted at intervals of 20 min, on which UV-Vis analysis was conducted to determine the concentration of RhB. The intensities of the characteristic absorption of RhB diminished with the irradiation time, indicating the decrease in RhB concentration (Figure 3a). Blue shifts of the absorption peaks from approximately 553 to 537 nm indicated a de-ethylation of RhB [38]. The C-t curve (C: concentrations of RhB, t: the irradiation time) implies the high degeneration rate of 87.82% after the irradiation time of 220 min (Figure 3b), which is higher than that of reduced grapheme oxide (RGO)@Ni foam [21] and comparable to that of crystalline catalysts such as {[(BiI_6_)I_13_]·2I_3_·(H-BPA)_4_}*_n_* [24]. Clearly, the {(*i*-PrV)[Ag_2_(SCN)_4_]}*_n_* photocatalyst presents the high efficiency of photocatalytic degradation of RhB in seawater. However, in the reference experiments without a catalyst or light irradiation, a very low degradation rate of RhB can be obtained (lower than 5.0%) (Figure 3b). Furthermore, this photocatalyst can retain its catalytic activity after three catalytic cycles, implying its good endurance (Figure 3c). The reusability of the photocatalyst was verified by PXRD measurement on the catalyst recovered from the mixture by the centrifugal method; this catalyst was washed with ether and dried under vacuum (Figure 3d). Clearly, judging from the PXRD patterns, which were identical to the initial patterns, the recovered sample can maintain its bulk phase. Previous work has proved that the photocatalytic reactions can be accelerated with the presence of salts, and metal ions such as Na^+^ and Mg^2+^ can enhance the photocatalytic efficiency of dyes [23].

**Figure 4:**
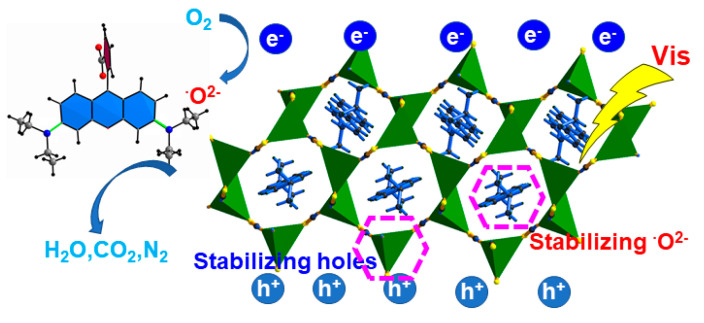
Photodegradation mechanism catalyzed by silver-thiocyanate/viologen photocatalyst.

The photodegradation mechanism of this work is similar to that of a viologen-containing photocatalyst [17], which is depicted in Figure 4. Due to the hydrophilicity of this photocatalyst, it can disperse into nano-particles in the seawater. Upon visible light irradiation, the {(*i*-PrV)[Ag_2_(SCN)_4_]}*_n_* particles can absorb photons of specific wavelengths due to their narrow energy gap of 2.65 eV, which will be excited to produce electron–hole pairs in the valence band (VB) and conduction band (CB). The holes (h^+^) in the VB will be captured by OH^−^ to generate ·OH, and the electrons in CB can couple with O_2_ in the air to generate superoxide radicals (O^2−^) [39]. In the viologen-bearing photocatalyst, the viologen can serve as a good electron acceptor, which can stabilize these highly reactive superoxide species, allowing ·O^2−^ to dominate in photocatalytic reactions. The holes could also be stabilized by electron-rich silver-thiocyanate anions. These photo-generated radicals can react with RhB, then form several intermediate products, and finally mineralize to CO_2_, H_2_O, and N_2_ and nitrate ions [40]. Overall, the photodegradation in this system is dominate by superoxide species. The high degradation efficiency is relative to its hydrophily, which can result in smaller nano-particles during the photodegradation of organic pollutants.

## 4. Conclusions

In summary, a stable viologen-bearing silver-thiocyanate-based photocatalyst, {(*i*-PrV)[Ag_2_(SCN)_4_]}*_n_*, was structurally determined and used in photocatalytic degradation of RhB in artificial seawater. The planar *i*-PrV^2+^ dications are confined in the cavities of the [Ag_2_(SCN)_4_]*_n_*^2*n*−^ layer with a 2D (6,3) mesh, which was further characterized by PXRD, UV-Vis, fluorescence, photocurrent, and water contact angle measurements. Good photocatalytic degradation performance on rhodamine B in artificial seawater was observed, during which the dye pollutant could be degraded, with a high degradation ratio of 87.82% in 220 min. This stable photocatalyst could be potentially used in the organic dye-type ocean pollutant treatment.

## Figures and Tables

**Table 1:** The crystal paraments and structure refined details for {(*i*-PrV)[Ag_2_(SCN)_4_]}*_n_*.

Empirical formula	C_10_H_11_AgN_3_S_2_
Formula weight	345.21
Temperature	296(2) K
Wavelength	0.71073 Å
Crystal system	Monoclinic
Space group	*P*2_1_/*c*
Unit cell dimensions	a = 9.970(6) Å
	b = 11.155(7) Å β = 96.713(8)°
	c = 11.536(7) Å
Volume	1274.2(14) Å^3^
Crystal size/mm	0.20 × 0.13 × 0.11
Z	4
Density (calculated)	1.799 mg/m^3^
Absorption coefficient	1.885 mm^−1^
F(000)	684
Theta range for data collection	2.06 to 25.05°
Index ranges	−11 ≤ h ≤ 11; −13 ≤ k ≤ 9;−12 ≤ l ≤ 13
Reflections collected	4052
Independent reflections	2199 [R(int) = 0.0171]
Reflections observed (>2sigma)	1891
Data Completeness	0.976
Absorption correction	None
Refinement method	Full-matrix least-squares on *F*^2^
Data/restraints/parameters	2199/0/147
Goodness-of-fit on F^2^	1.029
Final R indices [I > 2sigma(I)]	R_1_ = 0.0252 wR_2_ = 0.0549
R indices (all data)Largest diff. peak and hole	R_1_ = 0.0324 wR_2_ = 0.05850.446 and −0.626 e. Å^3^

**Table 2:** Selected bonds (Å) and angles (°) of for {(*i*-PrV)[Ag_2_(SCN)_4_]}*_n_*.

Ag(1)-S(1)	2.6471(19)	Ag(1)-S(2)	2.6050(13)
Ag(1)-N(2)	2.275(3)	Ag(1)-N(3)#1	2.262(3)
N(2)-Ag(1)-S(1)	102.56(7)	S(2)-Ag(1)-S(1)	114.16(4)
N(2)-Ag(1)-S(2)	107.38(9)	N(3)#1-Ag(1)-S(1)	105.50(8)
N(3)#1-Ag(1)-N(2)	123.69(11)	N(3)#1-Ag(1)-S(2)	104.00(9)
Symmtry codes: #1 *x*, −*y* + 1/2, *z*−1/2; #2 −*x* + 1, −*y*, −*z* + 1; #3 *x*, −*y* + 1/2, *z* + 1/2; #4 −*x* + 1, −*y*, −*z*			

**Table 3:** Hydrogen bonding details of {(*i*-PrV)[Ag_2_(SCN)_4_]}*_n_*.

D–H···A	D–H/Å	H···A/Å	D···A/Å	∠(D–H···A)/°	Symmetry Codes
C(1)-H(1B)···S(2)	0.960	2.965	3.894	163.13	*x* − 1, *y*, *z*
C(2)-H(2B)···S(1)	0.960	2.958	3.876	160.54	*x* − 1, *y*, *z*
C(7)-H(9)···S(1)	0.930	2.965	3.652	131.83	

## Data Availability

The original contributions presented in this study are included in the article. Further inquiries can be directed to the corresponding author.
